
JOURNAL INFORMATION
==============================
NLM Title Abbreviation: ScientificWorldJournal
Journal ID: ScientificWorldJournal
NLM Journal ID: 101131163
Journal ID: TSWJ

The Scientific World Journal

ISSN: 2356-6140
EISSN: 1537-744X
Publisher: Wiley

ARTICLE INFORMATION
==============================
PMCID: PMC5763693
PMID: 20563531
DOI: 10.1100/tsw.2010.108
Article ID: 425473
Article version: 1
Subjects: Directions in Science

Human Mesenchymal Stem Cells as Mediators of Breast Carcinoma Tumorigenesis and Progression

Rhodes Lyndsay V.
Burow Matthew E. *
Department of Medicine, Section of Hematology and Medical Oncology, Tulane University Health Science Center, New Orleans, LA, USA
*Matthew E. Burow: mburow@tulane.eduAcademic Editor: Martin Goette

Publication date: 2010
Electronic publication date: 2010 Jun 15
Volume: 10
First page: 1084
Last page: 1087
Received 2010 Mar 23; Revised 2010 May 6; Accepted 2010 May 11
Copyright: Copyright © 2010 Lyndsay V. Rhodes and Matthew E. Burow.
Copyright year: 2010
License: This is an open access article distributed under the Creative Commons Attribution License, which permits unrestricted use, distribution, and reproduction in any medium, provided the original work is properly cited.
License URL: https://creativecommons.org/licenses/by/3.0/

Keywords: mesenchymal stem cells, breast cancer, estrogen receptor, hormone independence, endocrine resistance, tumor microenvironment

Funding: Department of Defense Breast Cancer

==============================
Directions in Science
*TheScientificWorldJOURNAL* (2010) 10, 1084–1087
ISSN 1537-744X; DOI 10.1100/tsw.2010.108

TheScientificWorldJOURNAL
www.thescientificworld.com

### Human Mesenchymal Stem Cells as Mediators of Breast Carcinoma Tumorigenesis and Progression

Lyndsay V. Rhodes and Matthew E. Burow*

*Department of Medicine, Section of Hematology and Medical Oncology, Tulane University Health Science Center, New Orleans, LA*

**E-mail**: lvanhoy@tulane.edu; mburow@tulane.edu

*Received March 23, 2010; Revised May 6, 2010; Accepted May 11, 2010; Published June 15, 2010*

---

**KEYWORDS**: mesenchymal stem cells, breast cancer, estrogen receptor, hormone independence, endocrine resistance, tumor microenvironment

---

Breast cancer continues to be the most frequently diagnosed carcinoma in women in the U.S.[1,2] with one in eight (12%) women having the chance of developing some form of breast carcinoma over the course of their lifetime[3,4]. A tumor mass is composed of malignant cancer cells and nonmalignant benign cells. The benign cells include tumor endothelial cells, inflammatory cells, and stromal cells, as well as the extracellular matrix (ECM) that provides structural support to the malignant cells[5]. The tumor microenvironment has been shown to play an active part in tumorigenesis and cancer progression through structural support as well as secreted factors[6]. It is now understood that stromal fibroblasts within the tumor microenvironment can influence tumor cell activities, such as proliferation, survival, metastasis, and even tumor initiation[7,8,9,10,11].

The interaction between tumor cells and tumor stroma or microenvironment has been described as a two-way street, as the tumor cells influence the stroma via tissue remodeling and gene expression, and vice versa[5,12,13]. Tumor cells provide signals that stimulate *de novo* formation of bone marrow and ECM in order to provide stromal support to the growing tumor[14,15]. The host response to the establishment of tumor stroma closely mimics that of wound healing and scar development[16], leading to not only modified secreted proteins from tumor cells and stroma (direct action), but also the recruitment of other supporting cell types (indirect action), such as endothelial progenitor cells[7,17,18] and mesenchymal stem cells (MSCs)[19,20,21]. MSCs are multipotent progenitor cells that possess the ability to self-renew, while retaining the ability to differentiate into cell types of mesenchymal origin, including osteoblasts, chondrocytes, and adipocytes[22,23,24,25]. These cells are known contributors to tissue repair and wound healing[26]. Although MSCs are present in the bone marrow, small stores of MSCs can also be isolated throughout various tissues[27,28,29] that are proposed to act as reserves for wound repair and scar formation[30,31]. When tissue damage occurs, specific endocrine signals are released from the injury site, initiating MSC mobilization to the location of the damage[32,33,34].

It is thought that the tumor microenvironment mimics injury sites as "wound[s] that never heals"[16,35,36]. It has been proposed that the signals regulating increased turnover and proliferation of stromal cells in the tumor microenvironment may mediate engraftment of MSCs at the tumor site[36]. The effects mediated by MSCs were originally thought to involve stem cell differentiation or fusion

*Corresponding author.
©2010 with author.
Published by TheScientificWorld; www.thescientificworld.com events[37,38], however, subsequent observations proved that these cells can have sufficient effects without evidence of long-term engraftment at the site[39,40,41]. It has been suggested that MSCs home to tumor cells and surround the tumors without infiltrating them, indicating that any effects of the MSCs results from stromal factors and paracrine signaling[42]. MSCs secrete high levels of cytokine, chemokines, and growth factors basally, yet these secretion profiles can be changed depending on the culture conditions or microenvironment[43].

Because of the inherent ability of MSCs to home to tumor sites, the therapeutic potential for targeted therapy is an area of great possibility. However, many effects of MSCs on cancer cells seem to be cell-type specific, reinforcing the importance of tumor microenvironment and cell-to-cell communication[44]. Conflicting findings on the effects of MSCs on cancer led us to investigate the effects of MSCs on estrogen receptor–positive (ER+) breast tumorigenesis. In our recently published study, we demonstrated the effect of MSCs on primary breast tumor growth and the progression of these tumors to hormone independence[45]. Coinjection of bone marrow–derived MSCs enhanced primary tumor growth of the ER+, hormone-dependent breast carcinoma cell line MCF-7 approximately fourfold in the presence and twofold in the absence of estrogen in female ovariectomized immunocompromised mice. The most striking effect of MSCs on MCF-7 tumorigenesis was observed when cells were injected in the absence of estrogen and matrigel. Here we found an 80% tumor formation compared to 0% when MCF-7 cells were injected alone. These results demonstrated the ability of MSCs to stimulate hormone-independent growth of basally hormone-dependent cells, a progression that is associated with a more aggressive phenotype. These effects were found in conjunction with over a 10% increase in immunohistochemical staining of the progesterone receptor (PgR) in the MCF-7/MSC tumors as compared to MCF-7 control tumors. This increase in PgR expression indicates a link between MCF-7 cells and MSCs through ER-mediated signaling. Taken together, our data reveal the relationship between tumor microenvironment and tumor growth, and the progression to hormone independence. This tumor stroma-cell interaction may provide a novel target for the treatment of ER+, hormone-independent, and endocrine-resistant breast carcinoma.

In addition to work done in our lab, others have shown MSCs to be involved in other aspects of tumor formation and progression. In their 2007 paper, Karnoub and colleagues proposed that the metastatic traits of breast cancer cells are acquired through exposure of the epithelial cells to mesenchymal cells in the tumor-associated stroma[46]. It was suggested that MSCs provide paracrine signals to the tumor cells promoting metastasis via chemokine signaling (CCL5)[46], similar to tumor-associated stromal cells that have been shown to contribute to primary tumor growth *in vivo* via chemokine paracrine signaling[47]. Others have reported changes in proliferation as well as morphology, and cell-to-cell contact changes in breast carcinoma cells cultured in the presence of MSCs[48]. Furthermore, breast carcinoma cells cultured in the presence of MSCs have increased expression of epithelial-to-mesenchymal transition (EMT)–associated genes as demonstrated in a recent article by Martin et al.[49]. These findings suggest that not only do MSCs affect the growth response of tumor cells, but they also affect other biological processes as well, including the progression to hormone independence, EMT, and ultimately, metastatic potential.

Although approximately 70% of diagnosed breast carcinomas are ER+ and responsive to antiestrogen treatments (initially), the majority of these cases will progress to endocrine resistance and hormone independence. Understanding the mechanism(s) associated with this progression to a more aggressive and nonresponsive phenotype is paramount to the development and implication of novel treatments. The involvement of MSCs in the progression of breast cancer, including hormone independence, provides further insight and possibly new therapeutic targets in the treatment of advancing breast disease.

### ACKNOWLEDGMENTS

Original work by the authors was supported in part by the Department of Defense Breast Cancer Research Program (54551G1).

### REFERENCES

1. Early Breast Cancer Trialists' Collaborative Group (EBCTCG) (2005) Effects of chemotherapy and hormonal therapy for early breast cancer on recurrence and 15-year survival: an overview of the randomised trials. *Lancet* **365,** 1687–1717.
2. American Cancer Society (2009) Cancer Facts and Figures 2009. American Cancer Society, Atlanta.
3. Rahman, K.M., Sarkar, F.H., Banerjee, S., Wang, Z., Liao, D.J., Hong, X., and Sarkar, N.H. (2006) Therapeutic intervention of experimental breast cancer bone metastasis by indole-3-carbinol in SCID-human mouse model. *Mol. Cancer Ther.* **5,** 2747–2756.
4. Ries, L.A.G., Eisner, M.P., Kosary, C.L., Hankey, B.F., Miller, B.A., Clegg, L., Mariotto, A., Feuer, E.J., and Edwards, B.K. (2005) SEER Cancer Statistics Review, 1975-2002. National Cancer Institute, Bethesda, MD.
5. Bissell, M.J. and Radisky, D. (2001) Putting tumours in context. *Nat. Rev.* **1,** 46–54.
6. Bhowmick, N.A. and Moses, H.L. (2005) Tumor-stroma interactions. *Curr. Opin. Genet. Dev.* **15,** 97–101.
7. Orimo, A., Gupta, P.B., Sgroi, D.C., Arenzana-Seisdedos, F., Delaunay, T., Naeem, R., Carey, V.J., Richardson, A.L., and Weinberg, R.A. (2005) Stromal fibroblasts present in invasive human breast carcinomas promote tumor growth and angiogenesis through elevated SDF-1/CXCL12 secretion. *Cell* **121,** 335–348.
8. Bhowmick, N.A., Chytil, A., Plieth, D., Gorska, A.E., Dumont, N., Shappell, S., Washington, M.K., Neilson, E.G., and Moses, H.L. (2004) TGF-beta signaling in fibroblasts modulates the oncogenic potential of adjacent epithelia. *Science* **303,** 848–851.
9. Bhowmick, N.A., Neilson, E.G., and Moses, H.L. (2004) Stromal fibroblasts in cancer initiation and progression. *Nature* **432,** 332–337.
10. Hanahan, D. and Weinberg, R.A. (2000) The hallmarks of cancer. *Cell* **100,** 57–70.
11. Olumi, A.F., Grossfeld, G.D., Hayward, S.W., Carroll, P.R., Tlsty, T.D., and Cunha, G.R. (1999) Carcinoma-associated fibroblasts direct tumor progression of initiated human prostatic epithelium. *Cancer Res.* **59,** 5002–5011.
12. Hu, M. and Polyak, K. (2008) Molecular characterisation of the tumour microenvironment in breast cancer. *Eur. J. Cancer* **44,** 2760–2765.
13. Liotta, L.A. and Kohn, E.C. (2001) The microenvironment of the tumour-host interface. *Nature* **411,** 375–379.
14. Hasebe, T., Sasaki, S., Sugitoh, M., Ono, M., Saitoh, N., and Ochiai, A. (2001) Highly proliferative intratumoral fibroblasts and a high proliferative microvessel index are significant predictors of tumor metastasis in T3 ulcerative-type colorectal cancer. *Hum. Pathol.* **32,** 401–409.
15. Kuniyasu, H., Abbruzzese, J.L., Cleary, K.R., and Fidler, I.J. (2001) Induction of ductal and stromal hyperplasia by basic fibroblast growth factor produced by human pancreatic carcinoma. *Int. J. Oncol.* **19,** 681–685.
16. Dvorak, H.F. (1986) Tumors: wounds that do not heal. Similarities between tumor stroma generation and wound healing. *N. Engl. J. Med.* **315,** 1650–1659.
17. Li, H., Fan, X., and Houghton, J. (2007) Tumor microenvironment: the role of the tumor stroma in cancer. *J. Cell. Biochem.* **101,** 805–815.
18. Aghi, M., Cohen, K.S., Klein, R.J., Scadden, D.T., and Chiocca, E.A. (2006) Tumor stromal-derived factor-1 recruits vascular progenitors to mitotic neovasculature, where microenvironment influences their differentiated phenotypes. *Cancer Res.* **66,** 9054–9064.
19. Hombauer, H. and Minguell, J.J. (2000) Selective interactions between epithelial tumour cells and bone marrow mesenchymal stem cells. *Br. J. Cancer* **82,** 1290–1296.
20. Ritter, E., Perry, A., Yu, J., Wang, T., Tang, L., and Bieberich, E. (2008) Breast cancer cell-derived fibroblast growth factor 2 and vascular endothelial growth factor are chemoattractants for bone marrow stromal stem cells. *Ann. Surg.* **247,** 310–314.
21. Schichor, C., Birnbaum, T., Etminan, N., Schnell, O., Grau, S., Miebach, S., Aboody, K., Padovan, C., Straube, A., Tonn, J.C., and Goldbrunner, R. (2006) Vascular endothelial growth factor A contributes to glioma-induced migration of human marrow stromal cells (hMSC). *Exp. Neurol.* **199,** 301–310.
22. Haynesworth, S.E., Baber, M.A., and Caplan, A.I. (1992) Cell surface antigens on human marrow-derived mesenchymal cells are detected by monoclonal antibodies. *Bone* **13,** 69–80.
23. Mackay, A.M., Beck, S.C., Murphy, J.M., Barry, F.P., Chichester, C.O., and Pittenger, M.F. (1998) Chondrogenic differentiation of cultured human mesenchymal stem cells from marrow. *Tissue Eng.* **4,** 415–428.
24. Pittenger, M.F., Mackay, A.M., Beck, S.C., Jaiswal, R.K., Douglas, R., Mosca, J.D., Moorman, M.A., Simonetti, D.W., Craig, S., and Marshak, D.R. (1999) Multilineage potential of adult human mesenchymal stem cells. *Science* **284,** 143–147.
25. Qiao, C., Xu, W., Zhu, W., Hu, J., Qian, H., Yin, Q., Jiang, R., Yan, Y., Mao, F., Yang, H., Wang, X., and Chen, Y. (2008) Human mesenchymal stem cells isolated from the umbilical cord. *Cell Biol. Int.* **32,** 8–15.
26. Prockop, D.J. (1997) Marrow stromal cells as stem cells for nonhematopoietic tissues. *Science* **276,** 71–74.
27. Erices, A., Conget, P., and Minguell, J.J. (2000) Mesenchymal progenitor cells in human umbilical cord blood. *Br. J. Haematol.* **109,** 235–242.
28. Igura, K., Zhang, X., Takahashi, K., Mitsuru, A., Yamaguchi, S., and Takashi, T.A. (2004) Isolation and characterization of mesenchymal progenitor cells from chorionic villi of human placenta. *Cytotherapy* **6,** 543–553.

29. Tsai, M.S., Lee, J.L., Chang, Y.J., and Hwang, S.M. (2004) Isolation of human multipotent mesenchymal stem cells from second-trimester amniotic fluid using a novel two-stage culture protocol. *Hum. Reprod.* **19,** 1450–1456.
30. Horwitz, E.M., Prockop, D.J., Fitzpatrick, L.A., Koo, W.W., Gordon, P.L., Neel, M., Sussman, M., Orchard, P., Marx, J.C., Pyeritz, R.E., and Brenner, M.K. (1999) Transplantability and therapeutic effects of bone marrow-derived mesenchymal cells in children with osteogenesis imperfecta. *Nat. Med.* **5,** 309–313.
31. Kidd, S., Spaeth, E., Dembinski, J.L., Dietrich, M., Watson, K., Klopp, A., Battula, V.L., Weil, M., Andreeff, M., and Marini, F.C. (2009) Direct evidence of mesenchymal stem cell tropism for tumor and wounding microenvironments using in vivo bioluminescent imaging. *Stem Cells* **27,** 2614–2623.
32. Fox, J.M., Chamberlain, G., Ashton, B.A., and Middleton, J. (2007) Recent advances into the understanding of mesenchymal stem cell trafficking. *Br. J. Haematol.* **137,** 491–502.
33. Ramirez, M., Lucia, A., Gomez-Gallego, F., Esteve-Lanao, J., Perez-Martinez, A., Foster, C., Andreu, A.L., Martin, M.A., Madero, L., Arenas, J., and Garcia-Castro, J. (2006) Mobilisation of mesenchymal cells into blood in response to skeletal muscle injury. *Br. J. Sports Med.* **40,** 719–722.
34. Ruster, B., Gottig, S., Ludwig, R.J., Bistrian, R., Muller, S., Seifried, E., Gille, J., and Henschler, R. (2006) Mesenchymal stem cells display coordinated rolling and adhesion behavior on endothelial cells. *Blood* **108,** 3938–3944.
35. Studeny, M., Marini, F.C., Dembinski, J.L., Zompetta, C., Cabreira-Hansen, M., Bekele, B.N., Champlin, R.E., and Andreeff, M. (2004) Mesenchymal stem cells: potential precursors for tumor stroma and targeted-delivery vehicles for anticancer agents. *J. Natl. Cancer Inst.* **96,** 1593–1603.
36. Studeny, M., Marini, F.C., Champlin, R.E., Zompetta, C., Fidler, I.J., and Andreeff, M. (2002) Bone marrow-derived mesenchymal stem cells as vehicles for interferon-beta delivery into tumors. *Cancer Res.* **62,** 3603–3608.
37. Spees, J.L., Olson, S.D., Whitney, M.J., and Prockop, D.J. (2006) Mitochondrial transfer between cells can rescue aerobic respiration. *Proc. Natl. Acad. Sci. U. S. A.* **103,** 1283–1288.
38. Spees, J.L., Olson, S.D., Ylostalo, J., Lynch, P.J., Smith, J., Perry, A., Peister, A., Wang, M.Y., and Prockop, D.J. (2003) Differentiation, cell fusion, and nuclear fusion during ex vivo repair of epithelium by human adult stem cells from bone marrow stroma. *Proc. Natl. Acad. Sci. U. S. A.* **100,** 2397–2402.
39. Chopp, M., Zhang, X.H., Li, Y., Wang, L., Chen, J., Lu, D., Lu, M., and Rosenblum, M. (2000) Spinal cord injury in rat: treatment with bone marrow stromal cell transplantation. *Neuroreport* **11,** 3001–3005.
40. Horwitz, E.M., Gordon, P.L., Koo, W.K., Marx, J.C., Neel, M.D., McNall, R.Y., Muul, L., and Hofmann, T. (2002) Isolated allogeneic bone marrow-derived mesenchymal cells engraft and stimulate growth in children with osteogenesis imperfecta: Implications for cell therapy of bone. *Proc. Natl. Acad. Sci. U. S. A.* **99,** 8932–8937.
41. Iso, Y., Spees, J.L., Serrano, C., Bakondi, B., Pochampally, R., Song, Y.H., Sobel, B.E., Delafontaine, P., and Prockop, D.J. (2007) Multipotent human stromal cells improve cardiac function after myocardial infarction in mice without long-term engraftment. *Biochem. Biophys. Res. Commun.* **354,** 700–706.
42. Djouad, F., Bony, C., Apparailly, F., Louis-Plence, P., Jorgensen, C., and Noel, D. (2006) Earlier onset of syngeneic tumors in the presence of mesenchymal stem cells. *Transplantation* **82,** 1060–1066.
43. Haynesworth, S.E., Baber, M.A., and Caplan, A.I. (1996) Cytokine expression by human marrow-derived mesenchymal progenitor cells in vitro: effects of dexamethasone and IL-1 alpha. *J. Cell. Physiol.* **166,** 585–592.
44. Kan, I., Melamed, E., and Offen, D. (2005) Integral therapeutic potential of bone marrow mesenchymal stem cells. *Curr. Drug Targets* **6,** 31–41.
45. Rhodes, L.V., Muir, S.E., Elliott, S., Guillot, L.M., Antoon, J.W., Penfornis, P., Tilghman, S.L., Salvo, V.A., Fonseca, J.P., Lacey, M.R., Beckman, B.S., McLachlan, J.A., Rowan, B.G., Pochampally, R., and Burow, M.E. (2009) Adult human mesenchymal stem cells enhance breast tumorigenesis and promote hormone independence. *Breast Cancer Res. Treat.* [Epub ahead of print]
46. Karnoub, A.E., Dash, A.B., Vo, A.P., Sullivan, A., Brooks, M.W., Bell, G.W., Richardson, A.L., Polyak, K., Tubo, R., and Weinberg, R.A. (2007) Mesenchymal stem cells within tumour stroma promote breast cancer metastasis. *Nature* **449,** 557–563.
47. Hall, B., Andreeff, M., and Marini, F. (2007) The participation of mesenchymal stem cells in tumor stroma formation and their application as targeted-gene delivery vehicles. *Handb. Exp. Pharmacol*. **(180),** 263–283.
48. Fierro, F.A., Sierralta, W.D., Epunan, M.J., and Minguell, J.J. (2004) Marrow-derived mesenchymal stem cells: role in epithelial tumor cell determination. *Clin. Exp. Metastasis* **21,** 313–319.
49. Martin, F.T., Dwyer, R.M., Kelly, J., Khan, S., Murphy, J.M., Curran, C., Miller, N., Hennessy, E., Dockery, P., Barry, F.P., O'Brien, T., and Kerin, M.J. (2010) Potential role of mesenchymal stem cells (MSCs) in the breast tumour microenvironment: stimulation of epithelial to mesenchymal transition (EMT). *Breast Cancer Res. Treat.* [Epub ahead of print]

**This article should be cited as follows**:

Rhodes, L.V. and Burow, M.E. (2010) Human mesenchymal stem cells as mediators of breast carcinoma tumorigenesis and progression. *TheScientificWorldJOURNAL* **10,** 1084–1087. DOI 10.1100/tsw.2010.108.
